# Application of Ultrasound on Monitoring the Evolution of the Collagen Fiber Reinforced nHAC/CS Composites *In Vivo*

**DOI:** 10.1155/2014/418302

**Published:** 2014-04-14

**Authors:** Yan Chen, Yuting Yan, Xiaoming Li, He Li, Huiting Tan, Huajun Li, Yanwen Zhu, Philipp Niemeyer, Matin Yaega, Bo Yu

**Affiliations:** ^1^Department of Ultrasonic Diagnosis, Zhujiang Hospital of Southern Medical University, Guangzhou 510282, China; ^2^The Second Clinical Medical College of Southern Medical University, Guangzhou 510282, China; ^3^Key Laboratory for Biomechanics and Mechanobiology of Ministry of Education, School of Biological Science and Medical Engineering, Beihang University, Beijing 100191, China; ^4^Department of Orthopaedic surgery and Traumatology, Freiburg University Hospital, Freiburg, Germany; ^5^Department of Orthopedics, Zhujiang Hospital of Southern Medical University, Guangzhou 510282, China

## Abstract

To date, fiber reinforce scaffolds have been largely applied to repair hard and soft tissues. Meanwhile, monitoring the scaffolds for long periods *in vivo* is recognized as a crucial issue before its wide use. As a consequence, there is a growing need for noninvasive and convenient methods to analyze the implantation remolding process *in situ* and in real time. In this paper, diagnostic medical ultrasound was used to monitor the *in vivo* bone formation and degradation process of the novel mineralized collagen fiber reinforced composite which is synthesized by chitosan (CS), nanohydroxyapatite (nHA), and collagen fiber (Col). To observe the impact of cells on bone remodeling process, the scaffolds were planted into the back of the SD rats with and without rat bone mesenchymal stem cells (rBMSCs). Systematic data of scaffolds *in vivo* was extracted from ultrasound images. Significant consistency between the data from the ultrasound and DXA could be observed (*P* < 0.05). This indicated that ultrasound may serve as a feasible alternative for noninvasive monitoring the evolution of scaffolds *in situ* during cell growth.

## 1. Introduction

Cell-based bone tissue engineering has emerged as a promising alternative to traditional bone graft treatment [1]. Due to mineralized collagen fibers making up the microstructure of natural bone tissue [2], a biomimetic nanohydroxyapatite/collagen (nHA/Col) scaffold reinforced by mineralizing type I collagen fiber seems to be a very promising system for bone tissue engineering [3–5]. Hence the development of mineralized collagen fiber composites is in urgent need of a noninvasive, quantifiable, and systematic method to monitor the complex regenerate function and degradation process* in vivo *and in real time. Accurate* in vivo* data is needed for a complete understanding of the mineralized collagen fiber and guiding the scaffold design.

Developing a simple and easy-to-use method to monitor regeneration process of the scaffolds is critical for bone tissue engineering research. Current approaches for acquiring precise bone mineral density (BMD) value are mostly by dual-energy X-ray absorptiometry (DXA) or computed tomography (CT) [6]. However, for one thing, the scanning and image reconstruction procedures are of complex operations, in high consumption and with strong radiation. Thus it is difficult to meet the need of a long-term evaluation of dynamic tracking. For another, DXA and CT could only provide morphological information and are unable to achieve the exploration of bone microstructure, which is the determining factor of bone function independently of BMD [7, 8]. To date, measurements on bone constructs and some desired mechanical parameters mainly rely on destructive and time-consuming histological and biochemical assay [9]. The substantial animal use, low repeatability, and difficulty of* in vivo* examination limit its application. There are also some budding nondestructive technologies. MR elastography (MRE) could make an assessment of mechanical properties, but it is limited by a poor spatial resolution at 5 mm [10]. Optical coherence tomography (OCT), with high spatial resolution but low penetration capacity, was mainly used in evaluation of vascular scaffold currently [11]. Microcomputed tomography (*μ*CT) could evaluate the scaffolds systematically, but its use was limited by its high expense and equipment requirement [12].

Hence, there remains a significant need for noninvasive techniques to sequentially monitor the progress of tissue construct evolution* in vivo *without periodic animal sacrifice. Since the report about ultrasonic speed and attenuation in bone in 1975 [13], ultrasound was gradually developed to be a noninvasive, nonradiative diagnostic tool of bone. Physical parameters tested by ultrasound were capable of reflecting bone density, quality, and some other mechanical factors of cancellous bone [14, 15]. Consequently, ultrasonic technology has been widely used in analyzing children's bone condition and osteoporosis in recent years and reveals a promising application [16]. However, ultrasound is rarely reported to evaluate scaffolds in bone tissue engineering. Recently, ultrasound elasticity imaging (UEI) has been found to be an available tool for characterizing mechanical changes of the implanted scaffold with high resolution and substantial detecting depth, but it is at expenses of higher cost, more specific hardware, and not easily accessible to most research groups [17, 18]. In our study, we attempted to establish a noninvasive, comprehensive, and convenient bone repair monitoring system based on diagnostic ultrasound.

Appropriate scaffolds capable of providing suitable structural and biological constructs are of great importance for cellular ingrowth [19]. In our preliminary study, an injectable thermo sensitive hydrogel composite based on CS, HA, and Col was demonstrated with great biocompatibility and excellent osteogenesis performance [20, 21]. In current research, we reshaped it as a mineralized collagen fiber reinforced solid scaffold (nHAC/CS) to obtain a stable initial mechanical strength. Moreover, to explore how rBMSCs affect the bone repair process, we added rBMSCs into the scaffold and made the comparison with the simple nHAC/CS group. The ultimate goal is to innovatively excavate diagnostic ultrasound to monitor the real-time remodeling information for the long cultivation period of the two scaffold groups. Systematic* in vivo *indexes were extracted from ultrasonic images, such as bone mass, BMD, calcification rate, degradation rate, and uniformity of inner structure, and were compared with the analyzed indexes of DXA. The feasibility of diagnostic ultrasound was illustrated as a direct tool to evaluate the evolution of constructs online for tissue engineers.

## 2. Material and Method

### 2.1. Fabrication of Scaffold Materials

#### 2.1.1. Preparation of Thiolated Chitosan

Chitosan (800 mg) was dissolved in acetum solution (400 mL, 1%). Iminothiolane hydrochloride (80 mg) was added after stirring for 5 hours. The pH of the solution was adjusted to 6.0 by adding sodium hydroxide (5 M). Dialysis with hydrogen chloride (5 M) was repeated 3 times. Thiolated-chitosan sample was prepared after freeze-drying.

#### 2.1.2. Synthesis of nHAC/CS Scaffold

The synthesis of nHA/Col (nHAC) powder has been reported previously [22]. It was assembled with nanofibrils of mineralized collagen and sterilized by X-ray irradiation. Thiolated-chitosan sample (200 mg) was dissolved in sodium hydroxide (10 mL, 0.1 M) and nHAC powder (200 mg) was added into the solution. Then the solution was stirred and dispersed evenly by ultrasonic wave. We removed the solution into 96-well plates carefully, and the sample was freeze-dried at room temperature.

### 2.2. Cell Isolation and Culture

Bone marrow was obtained from 12-week-old male SD rats. Briefly, femurs were aseptically removed and broken. The Bone marrow was absorbed by an injector, and then rat mesenchymal stem cells (rBMSCs) were isolated to the culture flask after centrifugation at 1500 RPM for 5 min. The rBMSCs were cultured in DMEM/F-12 medium and allowed to adhere for 24 hours. Nonadherent cells were then removed. After that, the cells were cultured at 37°C in 95% humidity and 5% carbon dioxide, and the medium was changed regularly every 3 days. After 3 weeks, adherent cells were detached by trypsin-EDTA (0.5 to 0.2 g/L, Invitrogen) and used for the* in vivo* experiments.

### 2.3. Implantation Experiment in SD Rats

**Figure 1:**
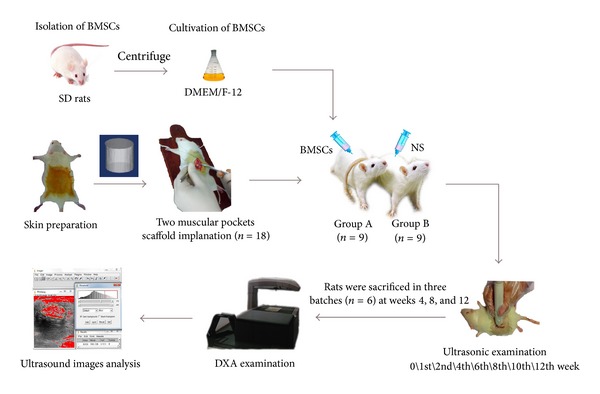
Schematic showing design of the study.

All the animals were operated in the light of the guidelines for animal experiments. In this study, 18 healthy SD female rats (150 g on average), supplied by the Animal Research Center of Guangdong Province, were divided into two groups equally (A, B). After induction with midazolam, the rats were anesthetized by the 0.3 mL/kg mixture of xylazine and ketamine (2 : 1). Then the rats were placed in the prone position, depilated, and sterilized from* arcus costarum* to hip joint. An incision was made close to erector spinae. We performed blunt dissection on superficial fascia and created three muscular pockets in the back. For each rat, two scaffolds of the same type were implanted. The columnar scaffold (nHAC/CS) was implanted with 0.5 mL concentrated solution of rBMSCs (5 × 10^6^) in the rats of group A, and in group B the same scaffold was implanted together with 0.5 mL normal saline (NS) as a control. The administration of antibiotics as prophylactic measure was carried out. All animals survived to the designated time without any major complications. The design of the study was displayed in Figure 1.

### 2.4. Ultrasonic Examination

Ultrasound images were taken with an ALOKA prosound *α*-10 premier diagnostic ultrasound system (1.1 mechanical index, 80 transmission gain) equipped with a 12 MHZ probe for all scans. Each group was performed a detection at week 0, week 1, week 2, week 4, week 6, week 8, week 10, and week 12. Rats were anesthetized in prone position with the inspection area exposed. Then we put the probe above the muscular pockets in order to observe the evolution of the scaffold constructs.

### 2.5. Ultrasound Images Analysis

The ultrasonic backscattered signal is displayed as a gray-scale array with values ranging from 0 to 255, and 0 denotes a negligible difference in resistance from the surrounding medium; the development of an ultrasound signal over time was interpreted as an increase in stiffness that may due to the solidifying development of materials. Gray-scale value, calcification rate, degradation rate, and homogeneous degree were measured and BMD was estimated by analyzing ultrasound images.

### 2.6. DXA

The rats were sacrificed in three batches (*n* = 6) at weeks 4, 8, and 12 with their affiliated tissue constructs harvested. And then each scaffold was scanned twice by a Lunar Prodigy DXA bone densitometer (GE Healthcare, Madison, WI, USA). BMD was used to evaluate the scaffolds' ability of heterotopic osteogenesis, which can be analyzed by LunarenCORE software (ver. 10.0, standard-array mode). All the measurements were executed by the same technologist who had received professional training.

#### 2.6.1. Gray-Scale Value

The gray-scale value (GV) was analyzed by measuring the mean GV of the implant area over time by the method of histogram echo intensity. The measurements from the six images were averaged together for each implant, reported as mean ± standard deviation.

#### 2.6.2. Calcification Rate and Degradation Rate

All images were analyzed by* ImageJ software* to measure calcification rate and degradation rate. Implant region was set as region of interest (ROI). According to the GV (“Min”-“Max”) of material tested in one hour after operation, GV ranging from “Max” to 255 was regarded as the region of calcification and the other was noncalcified region. Similarly gray-scale value ranging from 0 to “Min” and “Max” to 255 was regarded as the region of degradation and the other was no degradation region. Thus the calcification rate and degradation rate were estimated.

#### 2.6.3. Homogeneous Degree

Implant region was set as ROI. Homogeneous degree was calculated by applying the index “kurtosis” in* ImageJ*.

#### 2.6.4. BMD Estimation

According to the BMD and GV of radius, femur, tibia, pelvis, 7th cervical vertebrae, and 1st, 2nd, and 3rd lumbar vertebra in rats, regression curve was calculated. On the basis of this curve, BMD corresponding with each GV was estimated by the software of Origin 8.0. Finally, we carried out agreement analysis between estimated BMD and actual measured BMD by DXA.

### 2.7. Statistics

The correlation between two continuous variables, GV by ultrasound and BMD by DXA, was quantified with a Pearson correlation coefficient. Bland-Altman plots were used to assess the agreements between estimated BMD by ultrasound and actual measured BMD by DXA. The regression of the average and the difference between the two indicators were analyzed. All experimental data were reported as the mean ± standard deviation (*n* = 6). Levene homoscedasticity test and independent-samples *t*-test were used to identify any significant differences between the different groups. A *P* value of <0.05* and <0.01** was considered statistically significant. Statistical analyses were performed with SPSS19.0 software (SPSS Inc., Chicago, IL, USA).

## 3. Results and Discussion

### 3.1. The Mean Echo Intensity and Bone Formation

**Figure 2:**
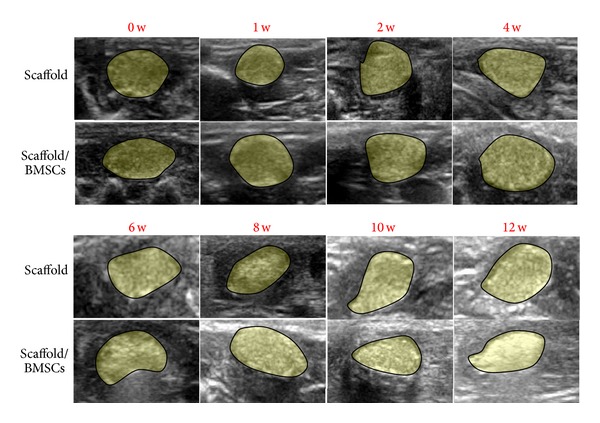
Ultrasound images of implanted scaffold over time, showing the evolution of constructs of the two groups (scaffold or scaffold/rBMSCs). The ROIs were signed by translucent yellow overlays.

**Figure 3:**
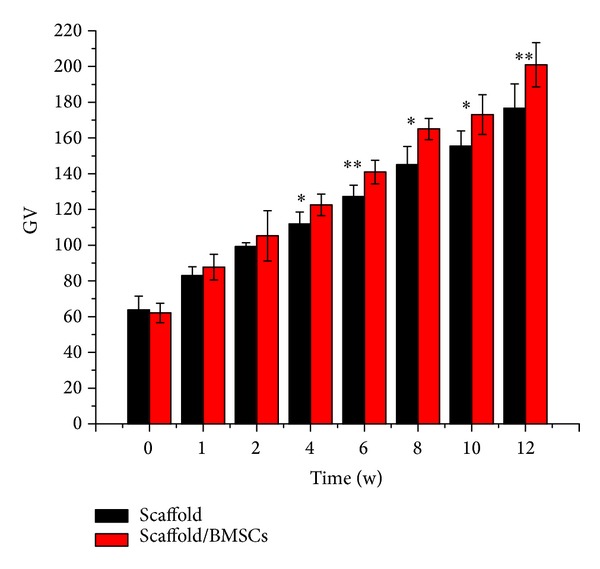
Ultrasound outcome indicating mean echo intensity (GV) of implant site for week 0, 1, 2, 4, 6, 8, or 10 postsurgery (*n* = 9 in each group). Results were expressed as mean ± SD (*n* = 9).

Ultrasound images of the implanted scaffolds with or without cells over time were displayed in Figure 2. It was obviously observed that the outline of implant was legible at each time point and the echo intensity of implanted site showed noticeable rise with time. Ultrasonic wave is largely attenuated through cancellous bone, and it was reported that attenuation of ultrasound propagating and acoustic velocity in bone is used widely for bone assessment [18, 23]. Gray-scale value (GV), which could digitize the mean echo intensity, is an indicator of acoustic impedance in implant site and has connection with medium density and sound velocity [24]. Thus, several researchers attempted to utilize GV to assess the tissue stiffness and mechanical properties [25–27]. Kreitz et al. [28] proposed GV as a good parameter to evaluate the collagen formation with a high correlation with hydroxyproline content (*r* = 0.98). In our study, to quantitatively analyse the changes of mean echo intensity among different time points and different groups, computer-assisted GV was measured in Table 1. A significant increase of GV over time was presented in Figure 3, probably representing the trend of bone mineral deposition and implanted scaffold calcification with respect to bone formation. The mean echo intensity of implant together with cells was enhanced from the fourth week (*P* < 0.05). The performance of rBMSCs may stimulate osteoblast differentiation as a result of GV level overtopping the control group. At 12 weeks after implanting, the GV of implant site jumped to 177 and 201, respectively, as high as the level of cancellous bone according to Table 4. The outcome indicated that the ultrasonic echo intensity (gray-scale brightness from images) could be a potential parameter assessing mechanical function* in vivo*. The GV indicator reflected a high consistency with the process of osteogenesis constructs, and the correlativity with BMD would be verified in the following sections.

### 3.2. The Process of Calcification and Degradation

In cell-based tissue engineering, the regeneration performance of scaffolds largely relies on their degradability. Current methods for quantifying degradation process are histological examination and direct sample measurements with animal sacrifice and scaffold destruction. Ultrasound is potentially to be applied as a noninvasive technique to obverse consequent scaffold degradation of the same specimen [29]. The degradation process results in different acoustical properties of the implantation site and could be detected by ultrasound as diminishing echo intensity [30, 31]. Chitosan was interfused in our scaffold to obtain better degradation property. The degradation of implanted scaffold could result in two conditions: for one, bone mineral deposition and calcified tissue ingrowth exactly at the degradation area; for another, the new bone formation is not as fast as the degradation of material and consequent cavitation or porosity could be observed* in situ*. Hence, the stable degradation rate of scaffold, which would exactly match the calcification rate, has a critical impact on the internal architecture and load-bearing capability. Calcification rate versus degradation rate could be a valuable indicator for scaffold assessment.

**Figure 4:**
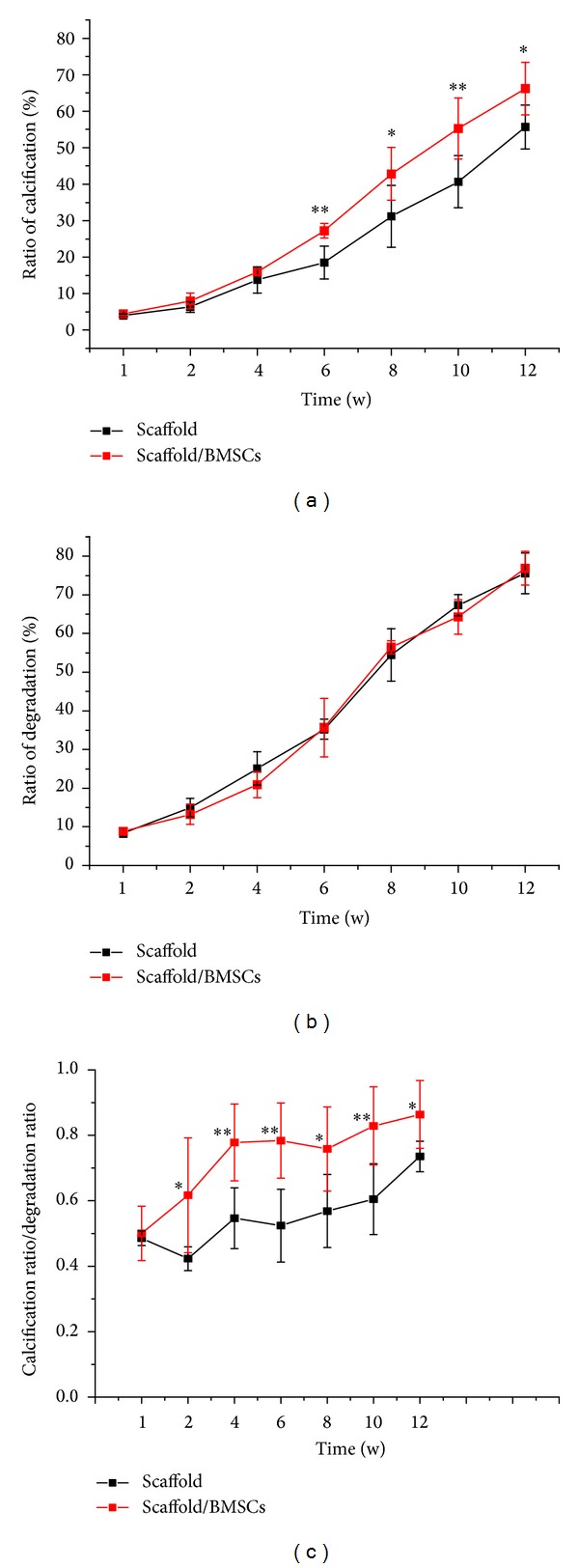
Regeneration property (a) and degradation property (b) of implant via ultrasound over time. Calcification rate versus degradation rate was calculated (c). Results were expressed as mean ± SD (*n* = 9); *P < 0.05 as compared to control group with no cells added.

The measurement results via ultrasound are listed in Table 2. The calcification rate of scaffolds with cells was much better than the control group (Figure 4(a)), while no statistically significant differences could be found between the two groups in degradation rate (Figure 4(b)), which indicated stem cells played an important role in calcification process and displayed no help in facilitating degradation. The scaffold/rBMSCs group possessed a higher value of the ratio (calcification rate versus degradation rate), which was a representative of better internal structure and more reliable mechanical support at its early stage. Ultrasound technique, as a noninvasive measure of monitoring the scaffold degradation and regeneration process, will greatly help tissue engineers improve the design process.

### 3.3. Homogeneous Degree of Implantation Area

Because the osteoporosis is thought to be largely determined by the combined effects of low bone content and poor microframework, the assessment of bone microstructure quality is of equal importance to BMD measure in bone tissue engineering. A systematic and ideal technique could assess the microstructure of scaffold noninvasively, which may have considerable impact on the mechanical indicator of fragility, stress, or strength. In our study, we attempted to utilize ultrasonic kurtosis coefficient to monitor the homogeneity of implantation area. Kurtosis is used to reflect the sharpness or flatness of the frequency distribution curve. Here, we calculated kurtosis coefficient as the concentration of GV near the mean as compared with the normal distribution. Therefore, the higher the value of kurtosis is, the more centralized the GV of implant site would be; that is, excessive calcification or excessive degradation region would not arise inside the implanted materials. Inversely, if the kurtosis coefficient of implant site is low or even negative, it is indicated that the frequency distribution curve of GV is relatively flat, which represents the heterogeneity of internal structure.

**Figure 5:**
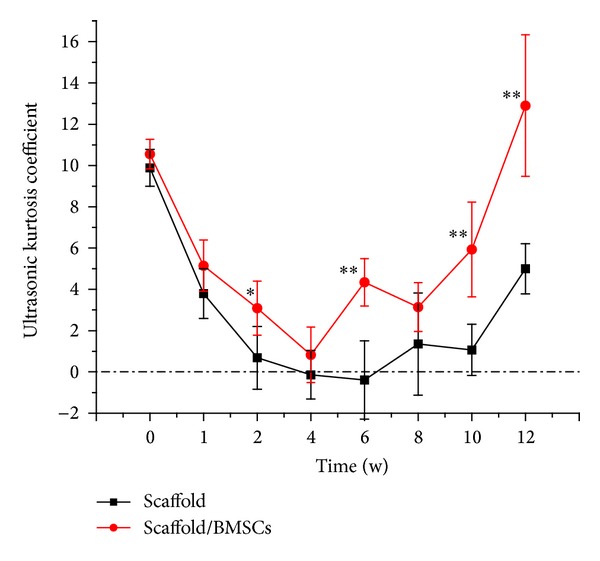
Graph of ultrasonic kurtosis coefficient of scaffold group and scaffold/rBMSCs group over time. Results were expressed as mean ± SD (*n* = 9); *P < 0.05 as compared to control group with no cells added.

Table 3 presented the kurtosis coefficient of GV in implant site at each time point. The homogeneous degree decreased in the first month after the implant operation and dropped to the lowest point at week 4 or so (Figure 5). It may be due to the rapid degradation rate in the early phase, which could give rise to the structural instability. Then with the constant calcification of scaffold, the voids were filled with new-born bone and the evenness index increased. Until 3 months, calcification was quite homogeneous and almost reached the internal morphology of cancellous bone as a result of a high kurtosis coefficient of GV. From the graph, the kurtosis coefficient of scaffold/rBMSCs group showed significantly higher than the control group up to 3 months after implanting (*P* < 0.01). It may be interpreted to be the effect of stem cells in modulating the microstructure of internal scaffold. Nevertheless, in the scaffold group without cells, kurtosis coefficient was less than 0 for approximately one month, which demonstrated a quite unstable internal structure with poor mechanical property and the implant area might cause more frequently collapse or distortion. Hence, it is of crucial importance to monitor the bone quality and material internal microstructure in real time in bone tissue engineering.

### 3.4. Comparison between the GV by Ultrasound and the BMD by DXA

**Figure 6:**
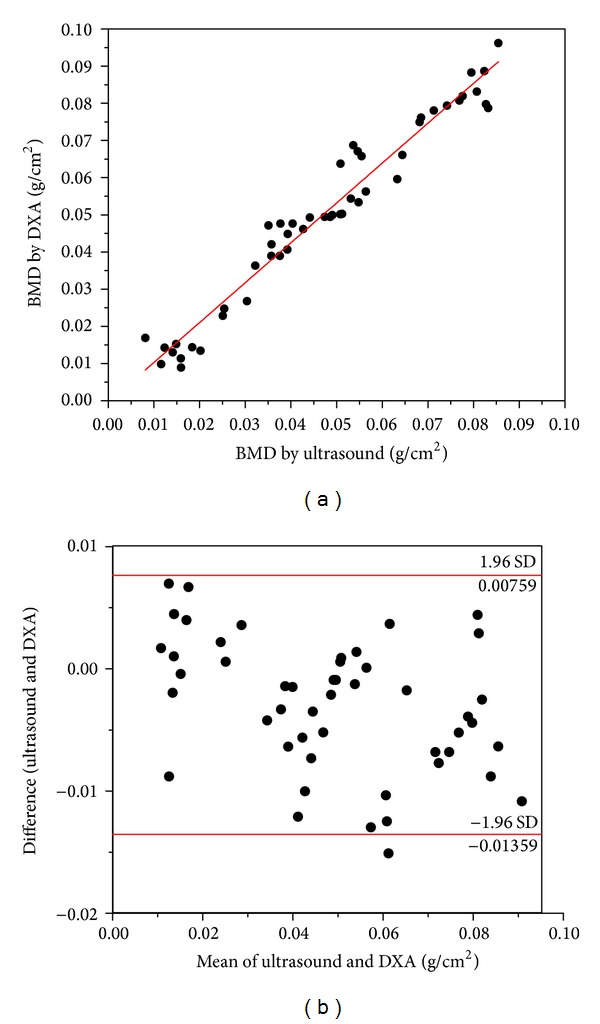
Linear regression plots (solid lines) of estimated BMD by ultrasound versus the measurements by DXA at different implant sites (a). Bland-Altman plots for agreement of data by ultrasound and DXA (b).

In recent researches, ultrasound technique has been gradually concerned with quantitative assessment of the scaffold constructs in animal studies [32, 33]. In order to demonstrate the feasibility of this approach for bone tissue engineering, some scholars made comparisons between ultrasound results and the traditional technique, such as histology or direct mechanical test, and a strong linear relationship was exhibited in their studies [17, 28, 34]. Measurement of BMD by DXA is generally considered to be the golden standard technique [35]. The relevant relationships between ultrasonic parameters and actual measurement by DXA was explored in our research. We first measured the radius, femur, tibia, pelvis, 7th cervical vertebrae, and 1st, 2nd, and 3rd lumbar vertebra of rats, and the data of GV by ultrasound and BMD by DXA was extracted in Table 4. The relationship between the two continuous variables was assessed with a bivariate correlation method (Pearson's test). Significant linear correlation between these two indicators was found to be the following: actual BMD = 5.34^−4^ GV−0.02 (*P* < 0.05; *r* = 0.96). According to the formula, we could estimate BMD of implant site based on the measured GV at different points in time (Table 1). Linear regression plot of estimated BMD by ultrasound parameters and direct measurements of BMD by DXA at different implant sites were presented in Figure 6(a). Then, Bland-Altman test was used to assess the agreements between the BMD measured by ultrasound and DXA. The regression of the mean and the difference was analyzed (Figure 6(b)). High consistency of estimated BMD and actual measured value was confirmed (*P* < 0.05). This is the key finding of our study and suggests that the ultrasonic techniques described in this paper can be a feasible alternative to invasively monitor the evolution of constructs online of tissue engineered scaffolds during cell growth.

## 4. Conclusions

This study established the validity of ultrasound as a noninvasive method to assess tissue transformation of the mineralized collagen fiber reinforced scaffold* in vivo* and in real time. We attempted to utilize ultrasound technology for providing accurate information of osteogenesis, degradation, and calcification process and homogeneity of the internal structure of the scaffolds without periodic animal sacrifice. An improvement in calcification rate and structural homogeneity with the growth of rBMSCs was observed by ultrasound, and the ultrasound findings matched direct BMD measurements by DXA distinctly. This study illustrated the potential of medical diagnosis ultrasound equipment for nondestructively monitoring the evolution of constructs online in bone tissue engineering. Also further research would be necessary to clarify this application before it is widely used.

## Figures and Tables

**Table 1:** Experimental data values for gray-scale value of the scaffold group and scaffold/rBMSCs group over time.

Time (w)	GV		*P* value
	Scaffold (mean ± SD)	Scaffold/BMSCs (mean ± SD)	
0	63.85 ± 7.7	62.07 ± 5.49	0.655
1	82.88 ± 5.04	87.73 ± 7.17	0.205
2	99.29 ± 2.13	105.23 ± 14.03	0.35
4	111.85 ± 6.76	122.53 ± 6.03	0.016*
6	127.23 ± 6.35	140.93 ± 6.57	0.004**
8	144.99 ± 10.31	165.08 ± 5.95	0.011*
10	155.44 ± 8.51	173.09 ± 11.14	0.016*
12	176.62 ± 13.75	200.99 ± 12.39	0.009**

*P < 0.05, **P < 0.01.

**Table 2:** Calcification and degradation characteristics of scaffolds at each time point.

Time (w)	Calcification rate (%) (mean ± SD)			Degradation rate (%) ( mean ± SD )			Calcification rate/degradation rate		
	Scaffold	Scaffold/BMSCs	*P* value	Scaffold	Scaffold/BMSCs	*P* value	Scaffold	Scaffold/BMSCs	*P* value
1	4.07 ± 0.4	4.43 ± 0.9	0.389	8.41 ± 1.1	8.84 ± 0.7	0.445	0.49 ± 0.02	0.5 ± 0.08	0.688
2	6.36 ± 1.4	7.97 ± 2.2	0.157	14.94 ± 2.4	13.21 ± 2.6	0.258	0.42 ± 0.04	0.62 ± 0.18	0.042*
4	13.76 ± 3.6	15.96 ± 0.8	0.201	25.1 ± 4.3	20.91 ± 3.3	0.09	0.55 ± 0.1	0.78 ± 0.12	0.004**
6	18.55 ± 4.5	27.27 ± 2	0.001**	35.21 ± 2.6	35.68 ± 7.5	0.888	0.52 ± 0.11	0.78 ± 0.12	0.003**
8	31.16 ± 8.5	42.83 ± 7.3	0.023*	54.44 ± 6.8	56.49 ± 1.7	0.504	0.57 ± 0.11	0.76 ± 0.13	0.021*
10	40.69 ± 7.2	55.25 ± 8.3	0.009**	67.32 ± 2.7	64.3 ± 4.4	0.636	0.61 ± 0.11	0.83 ± 0.12	0.007**
12	55.65 ± 6	66.23 ± 7.2	0.02*	75.59 ± 5.3	76.89 ± 4.3	0.649	0.74 ± 0.05	0.86 ± 0.1	0.02*

*P < 0.05, **P < 0.01.

**Table 3:** The kurtosis coefficient showing internal uniformity of scaffold at each time point.

Time (w)	Ultrasonic kurtosis coefficient		*P* value
	Scaffold (mean ± SD)	Scaffold/BMSCs (mean ± SD)	
0	9.89 ± 0.89	10.55 ± 0.72	0.187
1	3.8 ± 1.2	5.15 ± 1.24	0.084
2	0.69 ± 1.52	3.08 ± 1.31	0.016*
4	−0.14 ± 1.18	0.83 ± 1.35	0.961
6	−0.39 ± 1.9	4.34 ± 1.15	0.001**
8	1.35 ± 2.48	3.14 ± 1.18	0.142
10	1.07 ± 1.24	5.93 ± 2.3	0.002**
12	5 ± 1.21	12.9 ± 3.43	0.002**

*P < 0.05, **P < 0.01.

**Table 4:** Measured GV by ultrasound and BMD by DXA of radius, femur, tibia, pelvis, 7th cervical vertebrae, 1st & 2nd & 3rd lumbar vertebra in rats. Average values were calculated and recorded for each measurements (*n* = 5).

Detection part	GV by ultrasound	BMD (g·cm^−2^) by DXA
Radius	182	0.075
Femur	250	0.106
Tibia	197	0.088
Pelvis (ilium)	255	0.111
Vertebrae C7	190	0.089
Vertebrae LI	204	0.091
Vertebrae L2	213	0.098
Vertebrae L3	225	0.099
